# Lung Neutrophilia in Myeloperoxidase Deficient Mice during the Course of Acute Pulmonary Inflammation

**DOI:** 10.1155/2016/5219056

**Published:** 2016-02-21

**Authors:** Silvie Kremserova, Tomas Perecko, Karel Soucek, Anna Klinke, Stephan Baldus, Jason P. Eiserich, Lukas Kubala

**Affiliations:** ^1^Department of Free Radical Pathophysiology, Institute of Biophysics, Academy of Sciences of the Czech Republic, 61265 Brno, Czech Republic; ^2^Department of Experimental Biology, Faculty of Science, Masaryk University, 61137 Brno, Czech Republic; ^3^International Clinical Research Center, Center of Biomolecular and Cellular Engineering, St. Anne's University Hospital Brno, 65691 Brno, Czech Republic; ^4^Department of Cytokinetics, Institute of Biophysics, Academy of Sciences of the Czech Republic, 61265 Brno, Czech Republic; ^5^Heart Center, University of Cologne, 50937 Cologne, Germany; ^6^Department of Internal Medicine, School of Medicine, University of California, Davis, CA 95616, USA

## Abstract

Systemic inflammation accompanying diseases such as sepsis affects primarily lungs and induces their failure. This remains the most common cause of sepsis induced mortality. While neutrophils play a key role in pulmonary failure, the mechanisms remain incompletely characterized. We report that myeloperoxidase (MPO), abundant enzyme in neutrophil granules, modulates the course of acute pulmonary inflammatory responses induced by intranasal application of lipopolysaccharide. MPO deficient mice had significantly increased numbers of airway infiltrated neutrophils compared to wild-type mice during the whole course of lung inflammation. This was accompanied by higher levels of RANTES in bronchoalveolar lavage fluid from the MPO deficient mice. Other markers of lung injury and inflammation, which contribute to recruitment of neutrophils into the inflamed lungs, including total protein and other selected proinflammatory cytokines did not significantly differ in bronchoalveolar lavage fluid from the wild-type and the MPO deficient mice. Interestingly, MPO deficient neutrophils revealed a decreased rate of cell death characterized by phosphatidylserine surface expression. Collectively, the importance of MPO in regulation of pulmonary inflammation, independent of its putative microbicidal functions, can be potentially linked to MPO ability to modulate the life span of neutrophils and to affect accumulation of chemotactic factors at the inflammatory site.

## 1. Introduction

Inflammation is protective and vital to health, but when acute inflammation is unrestrained in amplitude or duration, it can lead to disease [1]. In most instances, the molecular and cellular events during acute inflammation are successful in limiting the inciting injury or infection and restoring tissue homeostasis. However, overwhelming inflammation can lead to fatal consequences. The lungs are generally the first to undergo failure, and this remains the most common cause of acute life-threatening pathologies such as septic shock and/or multiple organ failure induced mortality [2, 3]. Although acute inflammation is generally self-limited, alternate fates include abscess formation, fibrosis, or conversion to chronic inflammation [1].

While it has become clear that neutrophil granulocytes, the most abundant subpopulation of polymorphonuclear granulocytes, play a key role in pulmonary failure during sepsis and development of chronic inflammation, the mechanisms remain incompletely characterized [2, 4]. The progression and control of inflammation are significantly affected by mediators produced by leukocytes accumulated at the site of inflammation [1]. One of them is myeloperoxidase (MPO), an abundant hemoprotein of neutrophils, which is typically perceived to primarily mediate host defense reactions [5–7]. However, there is increasing evidence showing a novel regulatory role of MPO not directly related to host defense. MPO accumulated in the lungs could significantly modulate redox-sensitive cellular signaling pathways controlling inflammatory processes among others through the catabolism of nitric oxide (NO), induction of wide range of posttranslational modification of proteins, and modulation of metabolism of arachidonic and linoleic acid derived mediators [5–10].

However, the importance of MPO in various inflammatory conditions is questioned and is highly dependent on the type of the inflammatory model. Consistent with microbicidal function of MPO, the MPO deficient mice were more likely than the wild-type mice to be infected or die from infection employing various models, suggesting that the MPO-dependent oxidative system is important for host defense against fungi and bacteria [5, 6, 9]. However, the MPO importance varies by pathogen species [11]. In contrast, in the cases of inflammatory response to noninfectious stimuli or chronic inflammation in the absence of live pathogens, MPO can damage host tissue through the generation of oxidants. This has been observed in many chronic inflammatory diseases, including cardiovascular diseases, rheumatoid arthritis, and kidney diseases (see reviews [5–7, 9]). Thus, based on the assumption of detrimental effect of MPO during chronic inflammation, it can be expected that the inflammation should actually be reduced in MPO deficient mice. On the contrary, experimental data from various studies employing MPO deficient mice suggest that the MPO deficiency is connected with dysregulated inflammatory response. These studies show significant adverse effects of MPO deficiency in various inflammatory models induced by noninfectious stimuli including nonviable* Candida albicans* [12], experimental autoimmune encephalomyelitis [13], ischemic brain injury [14], lung dysfunction after allogeneic bone marrow transplantation [15], ultraviolet- (UV-) exposed skin inflammation [16], atherosclerotic lesions development [17], skin delayed-type hypersensitivity and antigen-induced arthritis [18], and different types of autoimmune renal diseases [19, 20]. These data support the suggestion of a protective, anti-inflammatory role of MPO in pathologies characterized by complex inflammatory response in the absence of infectious agent.

Here, a role of MPO in the regulation of acute lung inflammation and injury was evaluated. Model of acute airway inflammation induced by intranasal administration of lipopolysaccharide (LPS) was employed in both the wild-type and the MPO deficient mice. Various markers of inflammation and injury in lung tissues or lung lavage fluids were determined. Such LPS instillation is known to cause an acute inflammatory response with transient extravasation of primarily neutrophils in the airways [21–23]. The temporal course of inflammation was evaluated at different time points (8 h, 24 h, 48 h, and 72 h) following application of LPS. Our results indicate that MPO deficiency enhances neutrophilia during LPS-induced airway inflammation due to altered accumulation of proinflammatory cytokine RANTES and reduced cell death of MPO deficient neutrophils.

## 2. Materials and Methods

### 2.1. Animal Exposure to LPS

Male C57BL/6J wild-type and MPO deficient mice MPO_tm1lus (MPO deficient) (The Jackson Laboratory, USA) [8] both of age 12–16 weeks and of weight 25–30 g were subjected to brief anesthesia with ketamine-xylazine, and 50 *μ*L of LPS (from* Escherichia coli* serotype 055:B5, Sigma-Aldrich, USA) solution in phosphate-buffered saline (PBS) was instilled directly into their nostrils to reach a dose of 0.3 mg/kg LPS as described previously [23]. Previous studies demonstrated that a significant fraction of intranasally administered LPS will reach the lungs and that such instillation evokes an acute transient inflammatory response [21–23]. Mice in the control group received a similar volume of sterile PBS. The experiments were approved by the Animal Care Committee and were in accordance with the EU and NIH Guide for Care and Use of Laboratory Animals.

### 2.2. Collection of Bronchoalveolar Lavage Fluid (BALF)

At various time points after LPS instillation, mice were deeply anesthetized by intraperitoneal administration of ketamine-xylazine and blood was collected from the heart into heparinized syringes. The tracheae were cannulated, and the lungs were lavaged with 2 consecutive washes with 1 mL of PBS, which were pooled to a total recovered BALF of 1.6–1.8 mL. The BALF was used for the cell analysis after pelleting (5 min, 250 g, 4°C) or the cell-free BALF was obtained after centrifugation (4 min, 2 000 g, 4°C) and stored at −80°C until further analysis.

### 2.3. Determination of Inflammatory Cells in BALF and Their Differentiation Count

Total cell count in the collected lavage samples and total leukocyte count in blood were determined with a Coultre Counter Z1 after lysis of RBCs by Zap-Oglobin II lytic reagent (Coulter, USA). Cell differential counts were determined on blood smears or on slides after cytospin centrifugation stained with Giemsa stain (Diff-Quick, ThermoFisher Scientific, USA).

### 2.4. Inflammatory Cytokines and Total Proteins in BALF

To quantitate proinflammatory cytokine production in the lung tumor necrosis factor- (TNF-) *α*, monocyte chemoattractant protein- (MCP-) 1, Regulated on Activation, Normal T Cell Expressed and Secreted (RANTES), interleukin- (IL-) 6, IL-10, and IL-12 were determined in BALF by sandwich enzyme-linked immunosorbent assay (ELISA) using murine-specific commercial kits (Quantikine Mouse cytokine assay kit, R&D Systems, USA).

As a marker of epithelial injury and lung permeability, total protein concentration within BALF was measured with a Protein Assay Kit (Bio-Rad, USA) based on the method of Bradford, with bovine serum albumin as a standard.

### 2.5. Analysis of NO Production in Lungs

To quantitate the production of NO in the lungs, its metabolites nitrates/nitrites were measured in BALF by chemical reduction of nitrates and nitrites to NO in 120 mM vanadium (III) in 2 M HCl at 95°C and analysis by ozone-enhanced chemiluminescence (ANTEK Instruments Inc., Model 7020, USA).

### 2.6. Expression of CD11b on Neutrophil Surface

Cells were washed with PBS (5 min, 250 g, 4°C) and incubated with fluorescein-conjugated anti-mouse CD11b (Invitrogen, USA) antibodies at 4°C in the dark for 30 min, consequently washed with PBS (5 min, 250 g, 4°C), and analyzed using a BD FACSVerse*™* flow cytometer (BD Biosciences, USA) [24]. Cells incubated with appropriate isotype control were used to determine the nonspecific background signal. The data analysis was performed using Flowing Software (http://www.flowingsoftware.com).

### 2.7. Histological Analysis of Lungs

Following euthanasia, the lungs were instilled with OCT Compound (Sakura, The Netherlands). The lungs were subsequently removed and placed into OCT Compound and immediately frozen in liquid nitrogen. 7 *μ*m sections were stained with H&E stain and histological analysis was performed by light microscopy (10x objective). Series of pictures were obtained from the different types of samples that were evaluated and typical examples are represented.

### 2.8. Analysis of MPO Enzymatic Activity

Isolated cells were washed with PBS (5 min, 250 g, 4°C) and the remaining erythrocytes were lysed by hypotonic lysis using distilled water for 5 seconds. Cells were washed with PBS (5 min, 250 g, 4°C) and subsequently lysed in PBS with 0.1% Triton X-100 (Sigma-Aldrich, USA). Peroxidase activity was measured as the oxidation of tetramethylbenzidine (TMB, 2 mM, Sigma-Aldrich, USA) in 300 mM sodium acetate buffer (pH 5.4) in the presence of 0.075 mM hydrogen peroxide (H_2_O_2_) (Sigma-Aldrich, USA) within 20 min as described previously [24]. The formation of the reaction product was determined spectrophotometrically as the increase in absorption at 350 nm using an Infinite M200 microplate spectrofluorometer (Tecan, Switzerland).

### 2.9. Caspase 3 Activity Assay

Caspase 3 activity was analyzed as described previously [25]. Isolated cells were washed twice with PBS (5 min, 250 g, 4°C) and lysed (50 mM HEPES; 5 mM CHAPS; 5 mM DTT) on ice for 20 min and centrifuged (15 min, 15 000 g, 4°C). Lavage fluid was diluted 1 : 1 with 2x concentrated lysing buffer. The proteins present in supernatants were quantified using Coomassie® Protein Assay (Bio-Rad, USA) and diluted to an equal concentration. 5 *μ*g of protein samples was incubated in an assay buffer in parallels (20 mM HEPES; 2.5 mM CHAPS, 5 mM DTT, 2 mM EDTA) containing 50 *μ*M of caspase 3 (Ac-DEVD-AMC) substrate (Sigma-Aldrich, USA) at 37°C for 4 h. The level of fluorescence was determined using microplate reader (Infinite 200, Tecan, Switzerland; 360 nm excitation, 460 nm emission).

### 2.10. Phosphatidylserine (PS) Externalization (Annexin V/Propidium Iodide Assay)

The presence of cells with permeable membrane (dead cells) and cells with surface expression of PS was evaluated by flow cytometry using propidium iodide (PI) and Annexin V staining, respectively [26]. Cells were washed with PBS (5 min, 250 g, room temperature), resuspended in 100 *μ*L Annexin V binding buffer (10 mM HEPES/NaOH, pH 7.4, 140 mM NaCl, 2.5 mM CaCl_2_), and incubated for 15 min with 1 *μ*L Annexin V-FITC (Apronex Ltd., Czech Republic). PI (1 *μ*g/mL) was added 1 min before analysis. Flow cytometric analysis of the stained cells was performed using a BD FACSVerse flow cytometer (BD Biosciences, USA). The cell population for the analysis was gated using forward- versus side-scatter parameters to exclude any debris. Per sample, 10 000 cells were collected. Three different populations can be identified using this assay: intact viable cells are negative for both PI and Annexin V-FITC, apoptotic cells are positive for Annexin V-FITC but negative for PI, and dead cells positive for both Annexin V-FITC and PI. The data analysis was performed using Flowing Software (http://www.flowingsoftware.com).

### 2.11. DNA Fragmentation

3*∗*10^6^ cells isolated from BALF were washed with PBS (5 min, 250 g, 4°C) and DNA was isolated using the Invisorb Apoptosis Detection Kit II (Invitek; Invitek GmbH, Germany). Gel electrophoresis was performed in 1.5% agarose (Sigma-Aldrich, USA), using 1 kbp DNA ladder as a marker (ThermoFisher Scientific, USA). DNA was stained with ethidium bromide (Sigma-Aldrich, USA) and scanned on UV-transilluminator using the Scion Image software (Scion Corporation, USA).

### 2.12. Statistical Analysis

Statistical comparisons were analyzed with the Student *t*-test for pairwise-dependent samples. A *p* value equal to or lower than 0.05 was considered statistically significant. Data are presented as the means ± standard error of the mean (SEM). All statistical analyses were carried out with Statistica 10 (StatSoft, USA).

## 3. Results

### 3.1. The LPS-Induced Airway Neutrophilia

**Figure 1:**
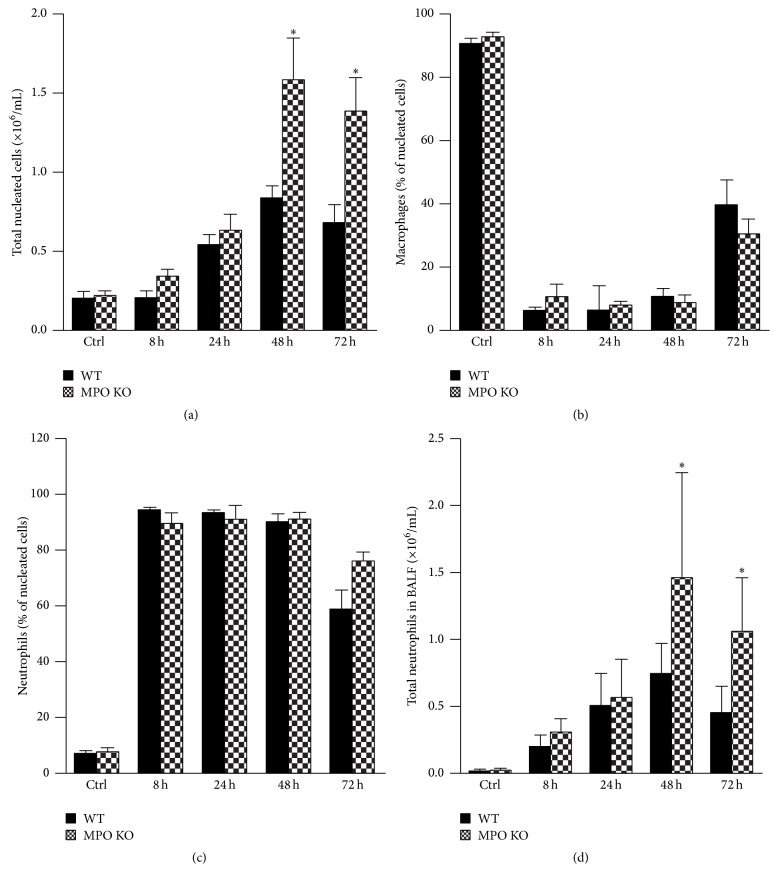
Acute lung inflammation induces accumulation of neutrophils in lungs. (a) Total nucleated cell counts, (b) relative count of airway macrophages, (c) relative count of neutrophils, and (d) total neutrophils in BALF collected at various times (8 h, 24 h, 48 h, and 72 h) after instillation of LPS (0.3 mg/kg) or PBS (control at time 0 h) in wild-type (WT; black bars) and MPO deficient (MPO KO; grey bars) mice. Values represent mean ± SEM from 8–10 mice with significant difference between WT and MPO KO mice (^*∗*^ *p* < 0.05).

**Figure 2:**
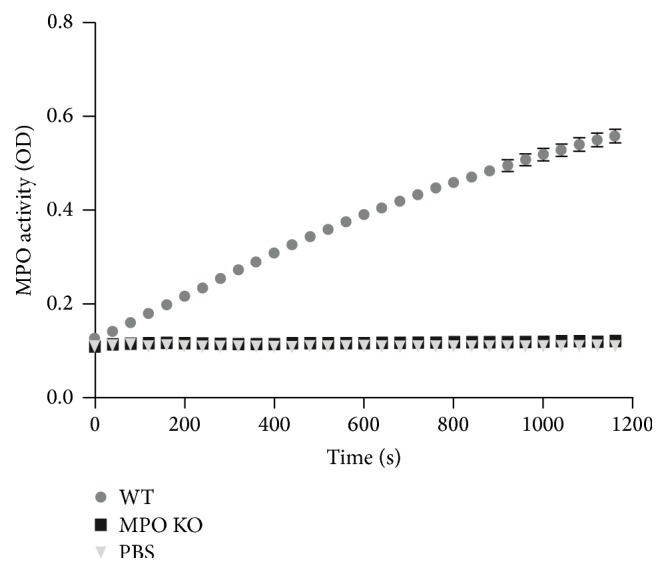
Peroxidase enzymatic activity of MPO in nucleated cells isolated from inflamed lungs of wild-type and MPO deficient mice. It was detected by spectrophotometry employing TMB. Data are presented as the increase in absorption at 350 nm and are expressed as mean ± SEM (*n* = 5).

Intranasal instillation of LPS (0.3 mg/kg) in the wild-type and the MPO deficient mice induced acute airway inflammation, characterized by an increase of the lung lavage cell numbers. This accumulation of cells in lungs was maximal 48 h after LPS instillation and declined thereafter (Figure 1(a)). As expected, while nucleated cells in BALF of untreated mice almost exclusively represented alveolar macrophages (Figure 1(b)), increased total numbers of nucleated cells in lung lavage of both wild-type and MPO deficient mice were almost exclusively due to neutrophils (Figure 1(c)) as assessed by Wright-Giemsa stain of cytospin samples. The number of lymphocytes, basophils, eosinophils, or other undetermined nucleated cells in BALF did not exceed 4% at any time point (data not shown). Interestingly, in contrast to expectations, the total numbers of nucleated cells in lavage were significantly lower in the wild-type mice compared to the MPO deficient mice at last two evaluated time points 48 h and 72 h after LPS instillation (Figure 1(a)). This was solely due to accumulation of neutrophils in lungs of the MPO deficient mice (Figure 1(d)), since as mentioned above, the differential count of cells in BALF did not differ significantly between the wild-type and the MPO deficient mice (Figures 1(b) and 1(c)). The determination of peroxidase activity in nucleated cells isolated from BALF of wild-type and MPO deficient mice clearly confirmed the absence of peroxidase activity in cells isolated from BALF of MPO deficient mice (Figure 2).

### 3.2. Abundance and Activation Status of Neutrophils in Peripheral Blood

**Figure 3:**
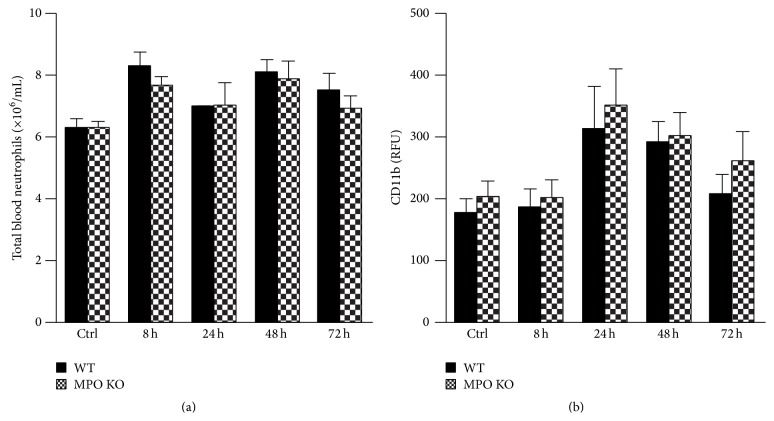
Total blood neutrophil count and surface expression of CD11b by neutrophils during the course of acute lung inflammation. (a) Total blood count of neutrophils was determined from total blood leukocytes count and their relative differentiation count in blood samples taken at various times (8 h, 24 h, 48 h, and 72 h) after instillation of LPS (0.3 mg/kg) or PBS (control at time 0 h) in both WT (black bars) and MPO KO (grey bars) mice. (b) Expression of surface CD11b receptor on blood neutrophils was determined by flow cytometer with a use of fluorescent labeled anti-CD11b monoclonal antibodies in blood samples described above. Results are expressed as relative fluorescence units (RFU). Values represent mean ± SEM from 8–10 mice.

Extravasation of neutrophils from peripheral circulation into the lungs is dependent on the number of neutrophils in the blood and activation status corresponding with a level of the surface expression of molecules (such as CD11b) involved in adhesion and migration of neutrophils through a vessel wall to extravascular space. Interestingly, in contrast to lung neutrophilia, the total neutrophil count in blood of wild-type and MPO deficient mice before and at different time points of LPS-induced lung inflammation did not significantly differ (Figure 3(a)). Similarly, the surface expression of CD11b did not differ significantly between blood neutrophils of the wild-type and the MPO deficient mice at any time point (Figure 3(b)). Further, the CD11b surface expression was also analyzed on cells isolated from BALF. Corresponding to peripheral circulation, the CD11b surface expression was the same in the wild-type and the MPO deficient animals (data not shown).

### 3.3. Alternations in the Lung Epithelial Cell Barrier Permeability and Increased Accumulation of NO Metabolites in BALF

**Figure 4:**
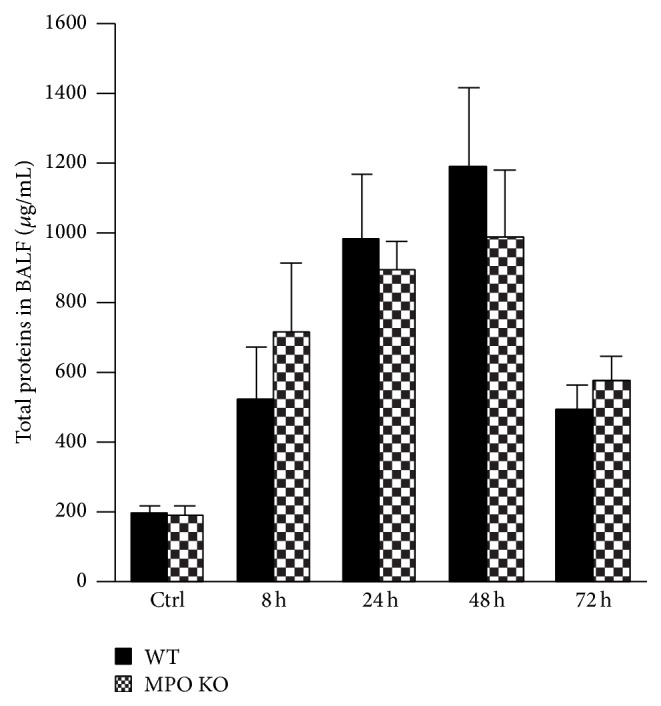
Total protein concentration during the course of acute lung inflammation. Total protein concentration in BALF at various times (8 h, 24 h, 48 h, and 72 h) after instillation of LPS (0.3 mg/kg) or PBS (control at time of 0 h) in both WT (black bars) and MPO KO (grey bars) mice. Values represent mean ± SEM from 8–10 mice.

**Figure 5:**
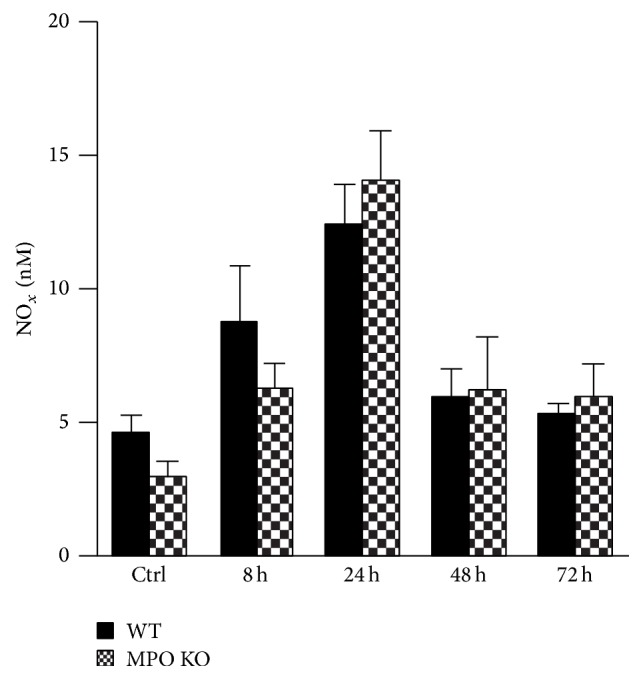
Accumulation of NO metabolites in BALF. Nitrates/nitrites in BALF at various time points (8 h, 24 h, 48 h, and 72 h) after instillation of LPS (0.3 mg/kg) or PBS (control at time 0 h) in both WT (black bars) and MPO KO (grey bars) mice. Values represent mean ± SEM from 8–10 mice.

**Figure 6:**
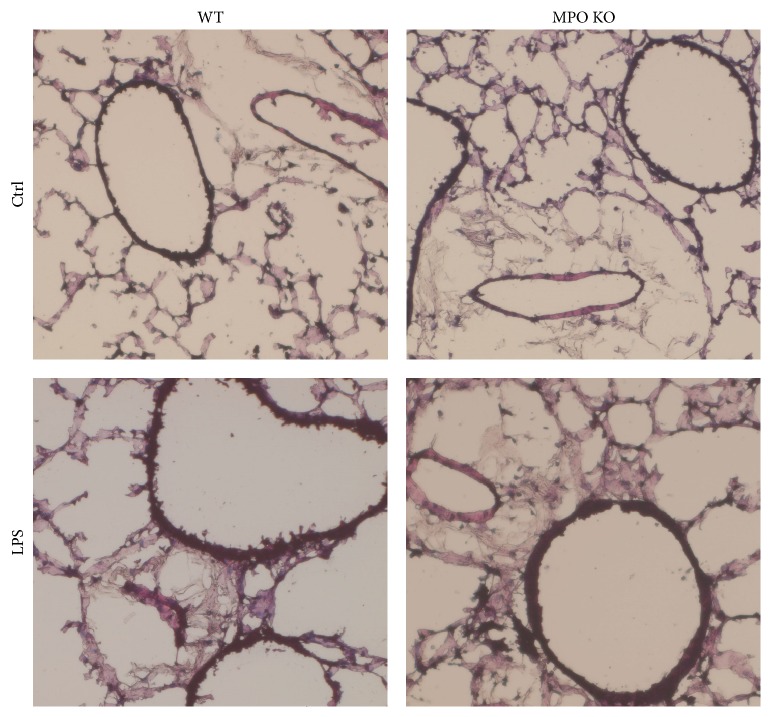
Lung histopathology. H&E staining of lung sections of WT and MPO KO mice 48 h after instillation of LPS (0.3 mg/kg) or PBS (ctrl).

Accumulation of neutrophils in lungs during the course of acute inflammation is also modulated by permeability of lung epithelium and capillaries of peripheral circulation. In our model, the increase in lung permeability, which is a marker of injury of lung epithelial cell barrier, was determined by measuring the total protein in BALF. The total protein concentration in BALF was significantly increased during acute lung inflammation with maximal levels at 48 h after LPS application (Figure 4). However, the total protein in BALF did not significantly differ between the MPO deficient and the wild-type mice at any time point. These findings suggest that significantly higher accumulation of neutrophils in the MPO deficient mice could not be explained by different permeability of lungs. The intranasal administration of LPS induced production of NO (determined as stable products nitrites and nitrates in BALF). The analysis revealed increased levels of NO production in both the MPO deficient and the wild-type mice, however, without significant differences between these groups (Figure 5). Finally, histological staining of lung sections showed no significant differences among lungs of wild-type and MPO deficient mice either in control groups or after 48 h of LPS instillation (Figure 6).

### 3.4. LPS-Induced Accumulation of Proinflammatory Cytokines in BALF

**Figure 7:**
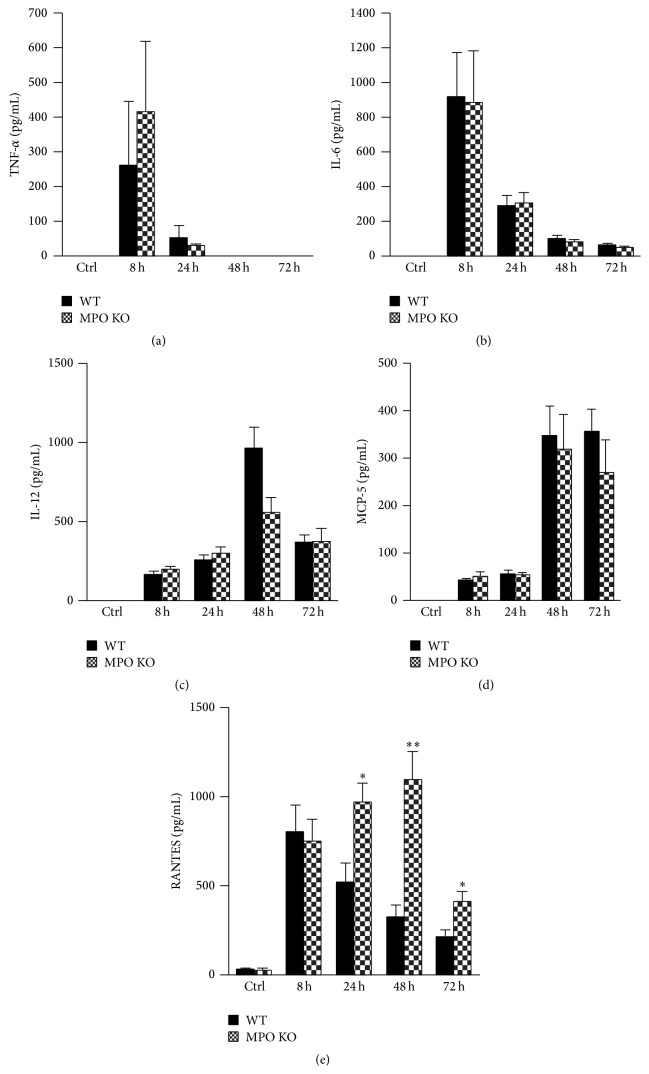
Levels of proinflammatory cytokines during the course of acute lung inflammation. (a) TNF-*α*, (b) IL-6, (c) IL-12, (d) MCP-5, and (e) RANTES concentrations in BALF were determined by commercial ELISA kits in samples described above. Values represent mean ± SEM from 8–10 mice with significant difference between WT and MPO KO mice (^*∗*^ *p* < 0.05; ^*∗∗*^ *p* < 0.01).

Corresponding with the course of inflammatory process, the proinflammatory cytokines were significantly increased in BALF after LPS instillation. TNF-*α* and IL-6 reached the maximal levels at the earliest time point after LPS instillation and decreased over the following days without significant differences between the MPO deficient and the wild-type mice (Figures 7(a) and 7(b)). IL-12 and MCP-5 reached maximal levels later, 48 h after the LPS instillation, and remained increased also at 72 h after LPS instillation but did not differ between MPO deficient and wild-type mice (Figures 7(c) and 7(d)). Levels of other potent chemoattractant RANTES increased from the first time point and in contrast to other evaluated cytokines were significantly higher in BALF of the MPO deficient mice compared to the wild-type mice (Figure 7(e)).

### 3.5. Increased Viability in BALF Cells from MPO Deficient Mice

**Figure 8:**
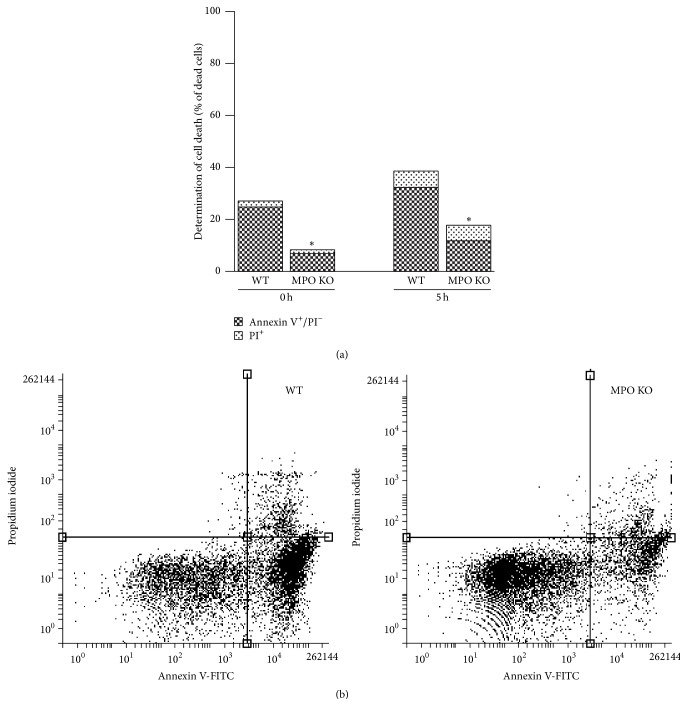
Determination of cell death in BALF cells. (a) Freshly isolated nucleated cells (1 × 10^6^ cells/mL) from BALF of WT and MPO KO mice treated for 36 h by intranasal application of LPS (0.3 mg/kg) or cells 5 h after isolation were evaluated by flow cytometry using Annexin V/PI staining. Values represent mean from 4 mice with significant difference between Annexin V^+^ cells in BALF from WT and MPO KO mice (^*∗*^ *p* < 0.05). (b) Representative scatterplots from flow cytometric analysis. Cells were classified as either viable (lower left quartile, Annexin V^−^/PI^−^) cells with detectable expression of PS (lower right quartile, Annexin V^+^/PI^−^) or dead cells with permeable membrane (upper left and right quartile, PI^+^).

**Figure 9:**
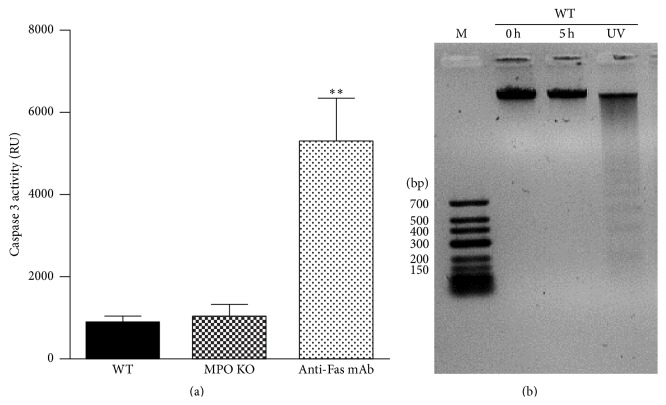
Determination of caspase 3 activity and DNA fragmentation. (a) Freshly isolated nucleated cells or cells 5 h after isolation (5 × 10^6^ cells/mL) from BALF of WT and MPO KO mice treated for 36 h by intranasal application of LPS (0.3 mg/kg) were lysed and caspase 3 activity was determined by enzymatic assay. Cells treated with anti-Fas antibody were used as a positive control. Values represent mean ± SEM from 8–10 mice with significant difference between WT or MPO KO mice and positive control (^*∗∗*^ *p* < 0.01). (b) DNA extracted from freshly isolated cells (0 h) or cells incubated* in vitro* for 5 h (5 h). Cells were isolated from BALF of WT mice treated for 36 h by intranasal application of LPS (0.3 mg/kg). UV-irradiated BALF cells were used as a positive control.

The accumulation of inflammatory cells can be mediated by alternated apoptosis of extravasated inflammatory cells. Thus, to further evaluate the observed phenomenon of higher accumulation of neutrophils in lungs of the MPO deficient mice after LPS instillation, the cells in BALF were examined for programmed cell death markers and for their ability to die after incubation* in vitro*. Interestingly, the cells isolated from BALF of the MPO deficient mice revealed a decrease of number of cells positive for Annexin V staining and cells positive for Annexin V/PI staining with permeable membrane (dead cells) (Figures 8(a) and 8(b)). After prolonged 5 h incubation of cells* ex vivo*, the number of Annexin V positive cells and dead cells increased in BALF cells from the wild-type mice whereas in the case of cells from the MPO deficient mice the number of these types of cells remained low (Figures 8(a) and 8(b)). Interestingly, there was only background caspase 3 activity either directly in BALF (data not shown) or in BALF cell lysate, either from the MPO deficient or from the wild-type mice (Figure 9(a)). Similarly, we did not observe any fragmentation of DNA either in freshly isolated cells or after prolonged 5 h incubation of cells* in vitro* (Figure 9(b)).

## 4. Discussion

In this study, the importance of MPO in the course of acute lung inflammation was evaluated. In contrast to our previous study showing MPO mediated potentiation of neutrophils extravasation into the site of inflammation in various other tissues, such as livers [27], herein the data show that the MPO deficient mice had significantly increased numbers of airway infiltrated neutrophils compared to the wild-type mice during the later course of lung inflammation induced by intranasal application of LPS. Interestingly, most markers characterizing status of lung injury and inflammation including total protein and selected proinflammatory cytokines in BALF did not significantly differ between the wild-type and the MPO deficient mice. The only exception was the BALF levels of RANTES that were increased in the MPO deficient mice. Interestingly, analysis of the cell death characterized by robust cell surface expression of PS revealed the significant delay of this process in BALF cells from the MPO deficient mice.

We did not observe any significant differences in the percentage of inflammatory cells in BALF between MPO deficient and wild-type mice. In agreement with our findings, Milla et al. did not observe any dissimilarities between wild-type and MPO deficient mice in BALF cell differentiation count in a model of transplantation induced lung inflammation [15]. The large numbers of neutrophils accumulated in the airspaces pose a major challenge for the host in resolving inflammation. Interestingly, the significantly more profound accumulation of leukocytes at the site of inflammation in MPO deficient mice was observed also by other authors using different types of murine inflammatory models. Takeuchi et al. reported that zymosan mediated inflammation led to greater neutrophil infiltration into the lungs of MPO deficient mice [28]. Further, this group showed that the MPO deficient mice that received nonviable* Candida albicans* showed more severe pneumonia with significantly higher numbers of alveolar neutrophils than wild-type mice [12]. Other studies revealed that MPO deficient mice exhibited accelerated lung dysfunction connected with increased number of inflammatory cells compared to wild-type mice after allogeneic bone marrow transplantation [15]. Also, recruitment of leukocyte into the peritoneum and glomerular accumulation of leukocytes including neutrophils were increased in pristine induced inflammation in MPO deficient mice [20]. On the other hand, Haegens and coauthors showed reduced inflammation in lungs of MPO deficient mice compared with wild-type mice despite employing a similar model of acute lung inflammation induced by intranasal LPS instillation [29]. However, these authors used different techniques for the estimation of lung infiltration and used higher dose of LPS compared to our study, which could result in a significantly higher level of inflammatory processes with a higher level of inflammation based injury. Under these conditions, other mechanisms responsible for the accumulation of neutrophils in lungs could predominate relative to the mechanisms suggested in our model. In general, the variability in results has to be examined in the context of the complexity of the lung inflammatory process when the infiltration and the accumulation of inflammatory cells in lungs are orchestrated by numerous factors [1, 3]. Thus, herein, a selection of these factors was determined to systematically evaluate this process to uncover factors that are affected by MPO and are connected or responsible for the observed alternations in lung inflammatory response in MPO deficient mice.

The extravasation of neutrophils into the site of injury is significantly affected by numbers of neutrophils in the peripheral circulation and their activation status. The employed MPO deficient mice were reported to have total white blood cell counts and differentials similar to the wild-type animals [17], which was confirmed in our study in both controls and during the course of acute lung inflammation. Similarly, other authors have not found any significant differences in the numbers of leukocytes and neutrophils between the wild-type and the MPO deficient mice in various inflammatory models [8, 15, 30]. Further, we did not observe any differences in the activation status of neutrophils determined based on the surface expression of the CD11b among the wild-type and the MPO deficient mice of either neutrophils in peripheral circulation or neutrophils obtained from lungs. The expression of CD11b was selected as a sensitive marker of neutrophil activation and also an important player of the neutrophil interaction with endothelium responsible for the neutrophil extravasation into the site of inflammation [31]. Further, the neutrophil influx into the lungs is affected by the permeability of the lung epithelium, which was determined by total protein concentration in BALF, primarily reflecting the leakage of albumin from lung capillaries of peripheral circulation into the alveolar space. However, this parameter did not differ significantly between the wild-type and the MPO deficient mice. In contrast, BALF protein levels in the model of transplantation induced lung inflammation were significantly higher in the MPO deficient compared with the wild-type mice [15].

The key role in the induction of neutrophil extravasation into the site of injury is played by proinflammatory cytokines, particularly with chemotactic potential. Corresponding to the course of inflammatory process, BALF levels of the proinflammatory cytokines were significantly increased after LPS instillation including two potent chemoattractants. MCP-5 increased later after LPS instillation but did not differ between the MPO deficient and the wild-type mice. In contrast, levels of RANTES were higher in BALF of the MPO deficient mice. RANTES is not a primary chemokine responsible for the extravasation of neutrophils at the site of inflammation; nevertheless, the correlation between levels of RANTES in lung lavage fluid and lung neutrophilia was presented also by the authors Lee et al. [32]. Thus, differences in levels of chemotactic RANTES could potentially contribute to an increased extravasation of neutrophils from blood periphery to the lungs and contribute to observed higher neutrophilia in the MPO deficient mice. Interestingly, authors from laboratory of Dr. Aratani reported that in model of zymosan induced lung inflammation the lavage from the MPO deficient mice contained significantly higher levels of macrophage inflammatory protein-2 (MIP-2) [28]. In their next study employing model of lung inflammation induced by nonviable* Candida albicans*, these authors showed that the MPO deficient mice had significantly increased production of MIP-2 and keratinocytes derived chemokine relative to the wild-type mice [12]. Furthermore, the MPO deficient mice had even significantly higher BALF concentrations of TNF-*α* and IL-1*β* than the wild-type mice [12]. Exploring the source of MIP-2, these authors showed that the MPO deficient neutrophils produce greater amount of MIP-2* in vitro* than do the wild-type neutrophils when stimulated with zymosan or* Candida albicans* [12, 28, 33]. Interestingly, the MIP-2 production was reduced when MPO was added to the MPO deficient neutrophils exogenously. These authors speculate that both the lack of hypochlorous acid and the accumulation of H_2_O_2_ due to MPO deficiency contribute to the upregulation of MIP-2 production in the mouse MPO deficient neutrophils. Similarly, in the model of transplantation induced lung inflammation the MPO deficient mice exhibited higher levels of TNF-*α* and the chemoattractant MCP-1 compared to the wild-type mice [15]. The importance of this phenomenon of higher levels of chemotactic factors in lungs of the MPO deficient mice for the observed lung neutrophilia was shown by Homme et al. when the neutralization of MIP-2* in vivo* significantly reduced neutrophil infiltration [12]. In context of our study, the presence of higher levels of RANTES could increase extravasation of neutrophils into the lungs and increased survival of neutrophils in lungs.

Another key factor affecting the accumulation of inflammatory cells in the inflamed lungs is the clearance of these cells from alveolar space. Thus, induction of regulated neutrophil cell death, apoptosis, is a critical event in the downregulation and resolution of inflammation [34, 35]. Therefore, the presence of dying cells in BALF from the MPO deficient and the wild-type mice was assessed by various methods including Annexin V staining, caspase 3 activity determination, and the DNA fragmentation. During apoptosis, PS residues on the inner leaflet of the cell membrane are externalized, providing a marker that can be detected by binding of Annexin V. Interestingly, significantly fewer cells positive for Annexin V and a delay in onset of PS externalization and cell permeability after* ex vivo* incubation were detected in BALF cells isolated from the MPO deficient mice. Substantial delay of the induction of cell death in the MPO deficient neutrophils strongly suggests possible mechanism for increased neutrophilia in the MPO deficient mice based on the accumulation of the MPO deficient neutrophils in the inflamed lungs. Interestingly, the defect of apoptotic induction in neutrophils from the MPO deficient mice was already suggested by other authors as well. Tsurubuchi et al. showed that the MPO deficient neutrophils stimulated with phorbol 12-myristate 13-acetate (PMA) or H_2_O_2_ underwent apoptosis significantly slower compared to normal neutrophils during interval up to 3 h of incubation [36]. However, in their other study employing zymosan induced lung inflammation they did not observe this phenomenon since they did not find an obvious difference in cell death between the wild-type and the MPO deficient neutrophils cultured for 6 h in the presence of zymosan [28]. Another model of the transplantation induced lung injury resulted in the increased number of inflammatory cells in BALF from the MPO deficient mice that was associated with suppressed apoptosis of BALF inflammatory cells [15].

Interestingly, in our study the observed neutrophil cell death is challenging to classify as conventional apoptosis since the dying neutrophils showed only one type of apoptotic marker, the expression of PS. Other markers of apoptosis, the caspase 3 activity, and DNA fragmentation were not present. In general, it can be suggested that the type of neutrophil cell death depends on the way of neutrophil activation. Interestingly, similar to our results Aratani and colleagues observed delayed PS externalization in PMA activated neutrophils from the MPO deficient mice that was not associated with caspase 3 activation [36, 37]. Moreover, Fadeel et al. showed the caspases activations in neutrophils treated by Fas ligand; however, it was absent in neutrophils stimulated with PMA [38]. In agreement with our results, DNA fragmentation was not observed in neutrophils incubated for 6 h alone or stimulated with PMA [39]. The PS surface exposure is known as one of the earliest markers of apoptosis and it precedes the morphologic appearances of apoptosis and changes in membrane permeability and the characteristic DNA fragmentation. Importantly, the PS exposure is a key mechanism by which apoptotic cells are recognized by macrophages, targeted for ingestion, and clearance from lungs [34, 35]. These results indicate that MPO probably participates in process of regulated cell death and contributes to clearance of neutrophils from lungs and reduction of airway neutrophilia. Taking into account our previous observations suggesting that MPO potentiate neutrophils to extravasate into site of inflammation in various other tissues, such as livers [27], we assume that in this case the higher number of neutrophils in the MPO deficient mice is not associated with the higher extent of LPS-induced neutrophil influx into the lungs. In contrast, we suggest that MPO deficiency is responsible for the suppressed cell death of lung infiltrating neutrophils.

Interestingly, the MPO effect on neutrophil presence in the lungs can be both dependent on and independent of MPO enzymatic activity. MPO enzymatic activity would be crucial also in the case of direct contribution of MPO as a cytotoxic agent that contributes to the induction of the death of cells accumulated in lungs. We can speculate that MPO enzymatic activity can decrease the formation of chemotactic mediators, as suggested by Tateno et al. [33]. Further, cytotoxic potential of MPO derived intermediates is also suggested to be important for the observed different sensitivity of MPO deficient neutrophils to apoptosis compared to the neutrophils of wild-type animals. MPO deficiency is suggested to be connected with suppressed regulated cell death in neutrophils by Milla et al. [15]. Furthermore, delayed PS externalization in PMA stimulated leukocytes from MPO deficient mice compared to leukocytes from wild-type mice was observed by Tsurubuchi et al. [36]. Likewise, pretreatment of neutrophils with the MPO inhibitor 4-ABAH blocked apoptosis induced by coactivation of neutrophils by TNF-*α* and H_2_O_2_ [40]. On the other hand, current data presented by Metzler et al. also suggest the importance of MPO independent of enzymatic activity in the specific regulated cell death NETosis [41]. Based on their observations, MPO is required independently of enzymatic activity for the release of proteases across intact membranes, which is key for the activation of specific proteases during NETosis. However, the importance of this newly suggested function of MPO independent of enzymatic activity has to be proven also in other types of regulated cell death that are relevant for the clearance of neutrophils from inflamed lungs.

In conclusion, these results suggest that neutrophil-derived MPO may play an important role in regulating the course of pulmonary inflammation, independent of its putative microbicidal functions. Because the MPO deficient neutrophils undergo delayed apoptosis* in vitro*, it is possible that these neutrophils remain alive longer at sites of inflammation. As a result, they would continue to release various ROS, inflammatory cytokines, and cytotoxic enzymes for a longer time, eventually resulting in tissue damage. However, further work evaluating the time course of appearance of apoptotic neutrophils is required to confirm the role of MPO in regulated cell death and to determine whether such defective functions of neutrophils are involved in the pathology of various inflammatory conditions. Knowledge gained from this research will help to determine more extensively the biological functions of MPO in inflammatory human lung disease and will aid in the development of potential pharmacological treatments for both acute and chronic lung injury.

## References

[1] Levy B. D., Serhan C. N. (2014). Resolution of acute inflammation in the lung. *Annual Review of Physiology*.

[2] Khadaroo R. G., Marshall J. C. (2002). ARDS and the multiple organ dysfunction syndrome: common mechanisms of a common systemic process. *Critical Care Clinics*.

[3] Cohen J. (2002). The immunopathogenesis of sepsis. *Nature*.

[4] Burns J. P. (2003). Septic shock in the pediatric patient: pathogenesis and novel treatments. *Pediatric Emergency Care*.

[5] Klebanoff S. J., Kettle A. J., Rosen H., Winterbourn C. C., Nauseef W. M. (2013). Myeloperoxidase: a front-line defender against phagocytosed microorganisms. *Journal of Leukocyte Biology*.

[6] Nauseef W. M. (2014). Myeloperoxidase in human neutrophil host defence. *Cellular Microbiology*.

[7] Nussbaum C., Klinke A., Adam M., Baldus S., Sperandio M. (2013). Myeloperoxidase: a leukocyte-derived protagonist of inflammation and cardiovascular disease. *Antioxidants and Redox Signaling*.

[8] Kubala L., Schmelzer K. R., Klinke A. (2010). Modulation of arachidonic and linoleic acid metabolites in myeloperoxidase-deficient mice during acute inflammation. *Free Radical Biology and Medicine*.

[9] Arnhold J., Flemmig J. (2010). Human myeloperoxidase in innate and acquired immunity. *Archives of Biochemistry and Biophysics*.

[10] Kubala L., Kolářová H., Víteček J. (2013). The potentiation of myeloperoxidase activity by the glycosaminoglycan- dependent binding of myeloperoxidase to proteins of the extracellular matrix. *Biochimica et Biophysica Acta (BBA)—General Subjects*.

[11] Aratani Y., Kura F., Watanabe H. (2000). Differential host susceptibility to pulmonary infections with bacteria and fungi in mice deficient in myeloperoxidase. *Journal of Infectious Diseases*.

[12] Homme M., Tateno N., Miura N., Ohno N., Aratani Y. (2013). Myeloperoxidase deficiency in mice exacerbates lung inflammation induced by nonviable *Candida albicans*. *Inflammation Research*.

[13] Brennan M.-L., Gaur A., Pahuja A., Lusis A. J., Reynolds W. F. (2001). Mice lacking myeloperoxidase are more susceptible to experimental autoimmune encephalomyelitis. *Journal of Neuroimmunology*.

[14] Takizawa S., Aratani Y., Fukuyama N. (2002). Deficiency of myeloperoxidase increases infarct volume and nitrotyrosine formation in mouse brain. *Journal of Cerebral Blood Flow and Metabolism*.

[15] Milla C., Yang S., Cornfield D. N. (2004). Myeloperoxidase deficiency enhances inflammation after allogeneic marrow transplantation. *The American Journal of Physiology—Lung Cellular and Molecular Physiology*.

[16] Komatsu J., Koyama H., Maeda N., Aratani Y. (2006). Earlier onset of neutrophil-mediated inflammation in the ultraviolet-exposed skin of mice deficient in myeloperoxidase and NADPH oxidase. *Inflammation Research*.

[17] Brennan M.-L., Anderson M. M., Shih D. M. (2001). Increased atherosclerosis in myeloperoxidase-deficient mice. *The Journal of Clinical Investigation*.

[18] Odobasic D., Kitching A. R., Yang Y. (2013). Neutrophil myeloperoxidase regulates T-cell-driven tissue inflammation in mice by inhibiting dendritic cell function. *Blood*.

[19] Odobasic D., Kitching A. R., Semple T. J., Holdsworth S. R. (2007). Endogenous myeloperoxidase promotes neutrophil-mediated renal injury, but attenuates T cell immunity inducing crescentic glomerulonephritis. *Journal of the American Society of Nephrology*.

[20] Odobasic D., Muljadi R. C., O'Sullivan K. M. (2015). Suppression of autoimmunity and renal disease in pristane-induced lupus by myeloperoxidase. *Arthritis & Rheumatology*.

[21] Okamoto T., Gohil K., Finkelstein E. I., Bove P., Akaike T., Van Der Vliet A. (2004). Multiple contributing roles for NOS_2_ in LPS-induced acute airway inflammation in mice. *The American Journal of Physiology—Lung Cellular and Molecular Physiology*.

[22] Poynter M. E., Irvin C. G., Janssen-Heininger Y. M. W. (2003). A prominent role for airway epithelial NF-*κ*B activation in lipopolysaccharide-induced airway inflammation. *The Journal of Immunology*.

[23] Viackova D., Pekarova M., Crhak T. (2011). Redox-sensitive regulation of macrophage-inducible nitric oxide synthase expression in vitro does not correlate with the failure of apocynin to prevent lung inflammation induced by endotoxin. *Immunobiology*.

[24] Kolarova H., Klinke A., Kremserova S. (2013). Myeloperoxidase induces the priming of platelets. *Free Radical Biology and Medicine*.

[25] Klinke A., Möller A., Pekarova M. (2014). Protective effects of 10-nitro-oleic acid in a hypoxia-induced murine model of pulmonary hypertension. *American Journal of Respiratory Cell and Molecular Biology*.

[26] Svihálková-Sindlerová L., Foltinová V., Vaculová A. (2010). LA-12 overcomes confluence-dependent resistance of HT-29 colon cancer cells to Pt (II) compounds. *Anticancer Research*.

[27] Klinke A., Nussbaum C., Kubala L. (2011). Myeloperoxidase attracts neutrophils by physical forces. *Blood*.

[28] Takeuchi K., Umeki Y., Matsumoto N. (2012). Severe neutrophil-mediated lung inflammation in myeloperoxidase-deficient mice exposed to zymosan. *Inflammation Research*.

[29] Haegens A., Heeringa P., van Suylen R. J. (2009). Myeloperoxidase deficiency attenuates lipopolysaccharide-induced acute lung inflammation and subsequent cytokine and chemokine production. *The Journal of Immunology*.

[30] Brennan M.-L., Wu W., Fu X. (2002). A tale of two controversies: defining both the role of peroxidases in nitrotyrosine formation in vivo using eosinophil peroxidase and myeloperoxidase-deficient mice, and the nature of peroxidase-generated reactive nitrogen species. *Journal of Biological Chemistry*.

[31] Kolaczkowska E., Kubes P. (2013). Neutrophil recruitment and function in health and inflammation. *Nature Reviews Immunology*.

[32] Lee C. S., Yi E. H., Lee J.-K. (2013). Simvastatin suppresses RANTES-mediated neutrophilia in polyinosinic-polycytidylic acid-induced pneumonia. *European Respiratory Journal*.

[33] Tateno N., Matsumoto N., Motowaki T., Suzuki K., Aratani Y. (2013). Myeloperoxidase deficiency induces MIP-2 production via ERK activation in zymosan-stimulated mouse neutrophils. *Free Radical Research*.

[34] Haslett C. (1999). Granulocyte apoptosis and its role in the resolution and control of lung inflammation. *American Journal of Respiratory and Critical Care Medicine*.

[35] Martin T. R., Nakamura M., Matute-Bello G. (2003). The role of apoptosis in acute lung injury. *Critical Care Medicine*.

[36] Tsurubuchi T., Aratani Y., Maeda N., Koyama H. (2001). Retardation of early-onset PMA-induced apoptosis in mouse neutrophils deficient in myeloperoxidase. *Journal of Leukocyte Biology*.

[37] Saito T., Takahashi H., Doken H., Koyama H., Aratani Y. (2005). Phorbol myristate acetate induces neutrophil death through activation of p38 mitogen-activated protein kinase that requires endogenous reactive oxygen species other than HOCl. *Bioscience, Biotechnology and Biochemistry*.

[38] Fadeel B., Åhlin A., Henter J.-I., Orrenius S., Hampton M. B. (1998). Involvement of caspases in neutrophil apoptosis: regulation by reactive oxygen species. *Blood*.

[39] Takei H., Araki A., Watanabe H., Ichinose A., Sendo F. (1996). Rapid killing of human neutrophils by the potent activator phorbol 12-myristate 13-acetate (PMA) accompanied by changes different from typical apoptosis or necrosis. *Journal of Leukocyte Biology*.

[40] Kanayama A., Miyamoto Y. (2007). Apoptosis triggered by phagocytosis-related oxidative stress through FLIPS down-regulation and JNK activation. *Journal of Leukocyte Biology*.

[41] Metzler K. D., Goosmann C., Lubojemska A., Zychlinsky A., Papayannopoulos V. (2014). A myeloperoxidase-containing complex regulates neutrophil elastase release and actin dynamics during NETosis. *Cell Reports*.

